# Effects of Compound Milk Oligosaccharides on Growth Performance, Immune Function, and Intestinal Health of Neonatal Suckling Piglets with Different Birth Weights

**DOI:** 10.3390/ani16193150

**Published:** 2026-10-08

**Authors:** Peng Zhang, Gang Tian, Daiwen Chen, Junning Pu, Ping Zheng, Jie Yu, Bing Yu

**Affiliations:** 1 Key Laboratory of Animal Disease-Resistance Nutrition, Institute of Animal Nutrition, Sichuan Agricultural University, No. 211 Huimin Road, Wenjiang District, Chengdu 611130, China; zhangpeng487x@163.com (P.Z.);; 2 Sichuan Chiyang Agricultural Development Co., Ltd., Room 2101, Floor 21, Unit 1, Building 3, No. 722, Middle Section of Yizhou Avenue, China (Sichuan) Pilot Free Trade Zone, Chengdu High-Tech Zone, Chengdu 610041, China

**Keywords:** colonic microbiota, compound milk oligosaccharides, growth performance, immune function, suckling piglets

## Abstract

This experiment was conducted to investigate the effects of compound milk oligosaccharides (CMO) on growth performance, immune function, and intestinal health of neonatal suckling piglets with different birth weights. Forty neonatal suckling piglets (20 piglets with normal birth body weight (NBW), 20 piglets with low birth body weight (LBW)) from 10 lactating sows with the same parity and litter size (2 normal birth weight piglets and 2 low birth weight piglets from each sow), were allotted to one of four treatments (10 replications with 1 piglet per treatment) in a 2 × 2 factorial design, including two birth body weight (normal or low birth weight) and two CMO doses (0 or 75 mg/d). All piglets were orally administered 2 mL CMO solution (37.5 mg/mL CMO, with a ratio of 3′ SL:2′ FL:6′ SL = 5:4:1) or equivalent volume saline daily, from birth until day 21 of age. At the end of the trial, eight piglets per treatment were used for terminal serum and intestinal measurements, and five piglets per treatment were used for colonic microbiota analysis. As expected, NBW piglets had higher body weight and average daily gain than LBW littermates (*p* < 0.05), accompanied by a lower colonic abundance of *Escherichia-Shigella* (*p* < 0.05). More importantly, oral CMO administration from birth to weaning improved body weight on days 14 and 21, average daily gain, jejunal villus height, and the villus height-to-crypt depth ratio, decreased serum IL-1β, IL-6, and IL-10 concentrations, and inhibited ileal mRNA expression of *IL-1β* and *MyD88* in piglets of both birth weight groups. In addition, CMO markedly downregulated ileal *NF-κB* expression in LBW piglets (*p* < 0.05). Interaction analysis indicated that the interaction between birth weight and CMO supplementation specifically decreased ileal *TLR4* gene expression, reduced colonic observed species, Chao 1 and ACE indices, and enriched colonic *Parabacteroides* in LBW piglets (*p* < 0.05). In summary, these findings demonstrated that a daily oral dose of 75 mg CMO improved growth performance and intestinal health in suckling piglets, and the underlying mechanisms may be closely associated with alleviation of intestinal inflammation, improvement of intestinal morphology, and modulation of gut microbial balance.

## Simple Summary

Milk oligosaccharides (MO) are recognized as prominent bioactive glycans in mammalian colostrum and mature milk, representing the third most abundant fraction after lactose and lipids. They exhibit multiple physiological activities, and the suckling period serves as a critical window phase for piglets to acquire MO. In this study, normal and low birth weight suckling piglets were used to investigate the effects of administration of compound milk oligosaccharides (CMO) on growth performance, immune function, and intestinal health, as well as the underlying mechanisms. The results demonstrated that oral administration of CMO improved growth performance and intestinal health in suckling piglets. These findings are expected to promote the development of novel milk replacers or specialized feed specifically designed for suckling piglets.

## 1. Introduction

In recent years, high-prolific sow genotypes have been widely adopted in commercial production, with a marked increase in litter size. In practice, litter sizes of 18–20 piglets per parity have become fairly common [1,2,3]. However, this enhanced prolificacy also brings about a series of challenges, including prolonged farrowing duration, which raises the risk of hypoxia and dystocia for neonatal piglets; reduced average birth weight, vitality, and within-litter uniformity; especially intensified competition for colostrum and milk intake. Insufficient maternal milk intake not only compromises passive immunity acquisition but also retards intestinal maturation and, in severe cases, increases pre-weaning mortality [4,5]. These adverse effects ultimately reduce the annual number of weaned piglets per sow and may negatively affect subsequent growth performance of the offspring. Previous studies have indicated that the increase in reproductive performance of high-prolific sows is not accompanied by a commensurate rise in total milk yield, implying that the per-piglet actual milk nutrient intake is potentially limited [6]. Therefore, supplementing the offspring of high-prolific sows with milk replacers may be an effective strategy to improve pre-weaning survival rate and weaning weight. However, research on the identification and screening of key functional ingredients in milk replacers remains scarce.

Breast milk is regarded as the gold standard of mammalian nutrition, providing essential nutrients for the growth and development of neonates. Studies on milk components have revealed that milk oligosaccharides (MOs) are important functional components, with their content being second only to lactose and fat [7,8,9]. MOs are typically composed of 3 to 10 monosaccharides linked by glycosidic bonds. The monosaccharide units include D-glucose, D-galactose, N-acetylglucosamine, L-fucose, N-acetylneuraminic acid, and N-glycolylneuraminic acid, which are linked by various glycosidic linkages [10,11]. Based on their molecular structures, MOs can be broadly classified into three categories: neutral fucosylated, neutral non-fucosylated, and sialylated MOs. Representative neutral fucosylated MOs include 2′-fucosyllactose (2′-FL) and 3′-fucosyllactose (3′-FL). Common neutral non-fucosylated MOs include lacto-N-tetraose (LNT), lacto-N-neotetraose (LNnT), and 3′-galactosyllactose (3′-GL). Sialylated MOs mainly consist of 3′-sialyllactose (3′-SL) and 6′-sialyllactose (6′-SL), etc. [12]. The nutrient profile of mammalian milk changes dynamically during lactation, which is thought to be associated with meeting the changing nutritional needs of neonates at different developmental stages; generally, MOs contents are higher in colostrum than in mature milk [13]. Human milk is particularly rich in MOs, with concentrations around 20–25 g/L in colostrum and 5–20 g/L in mature milk [13]. The composition of MOs also varies among species [14]. In human milk, the identified MOs are predominantly fucosylated neutral structures [12], whereas in porcine milk, sialylated structures are the major forms [9].

Studies have demonstrated that MOs exhibit multiple physiological activities, including immunomodulatory effects [15], prevention of pathogen adhesion to the intestinal epithelium [16,17,18], antiviral activity [19,20,21,22], protection against necrotizing enterocolitis [23], improvement of intestinal barrier function [24], regulation of the gut microbiota [25], and promotion of neural development [26,27,28,29]. It was reported that healthy full-term infants who received a 2′-FL-supplemented formula from day 5 after birth to 6 weeks of age, had plasma concentrations of pro-inflammatory cytokines (IL-1β, IL-6, and TNF-α) comparable to those of breastfed infants and significantly lower than those fed a conventional formula [30]. Additionally, this supplementation was associated with an increased proportion of apoptotic activated CD8^+^ T cells and reduced inflammatory cytokine expression in activated monocytes [30]. Using a neonatal piglet model infected with rotavirus, Li et al. [31] found that MOs could protect piglets from rotavirus infection and shorten diarrhea duration by inhibiting viral replication, enhancing mucosal Th1 and Th2 cytokine responses, and modulating gut microbial composition and its metabolism. These findings indicate that MOs play an important role in immune regulation in newborn animals. Beyond immunomodulation, MOs also effectively improve the growth performance of young animals. Dietary supplementation with sialyllactose was shown to improve intestinal morphology, enhance intestinal barrier function, and increase average daily gain in weaned piglets challenged with enterotoxigenic *Escherichia coli* (ETEC) [32]. Li et al. [25] reported that oral administration of sialyllactose to suckling piglets improved growth performance, which was closely associated with enhanced immune function, increased antioxidant capacity, and improved intestinal barrier integrity. Moreover, milk oligosaccharides resist upper gastrointestinal digestion and reach the colon intact, where they act mainly as prebiotics to modulate gut microbiota. Most MOs are fermented by beneficial bacteria, including *Bifidobacterium* and *Bacteroides*, producing postbiotics and SCFAs (acetate, propionate, and butyrate) [33,34]. SCFAs are the primary energy source for colonic epithelial cells and exert protective effects against pathogenic infection while improving intestinal barrier function [35,36,37]. Although a small portion of MOs may be directly utilized by colonocytes, bacterial fermentation and SCFA production represent the main pathway through which MOs contribute to colonic energy supply and intestinal health. In an *in vitro* study, Perdijk et al. [34] found that sialyllactose and galacto-oligosaccharides (GOS) differentially modulated the microbial community composition by promoting the proliferation of *Bacteroides* and *Bifidobacteria*, respectively, leading to significant changes in SCFA profiles. Furthermore, dietary sialyllactose supplementation significantly increased the counts of *Lactobacillus* and *Bacillus* in the colon of suckling piglets [25].

At present, research on MOs has mainly focused on the structural characterization, content analysis, and functional effects of individual MO monomers. For instance, individual sialyllactoses, such as 3′-SL and 6′-SL, have been shown to improve growth performance, intestinal morphology, immune function, and intestinal barrier integrity in weaned and suckling piglets [25,32]. In addition, 2′-FL and 6′-SL can suppress TLR4 signaling and alleviate intestinal inflammation [38,39], and sialyllactose can modulate gut microbiota composition [25,34]. However, the effects of a specific compound MO mixture (3′-SL:2′-FL:6′-SL = 5:4:1) on suckling piglets remain poorly understood. In particular, it is unknown whether this mixture can improve growth performance, immune function, and intestinal health in suckling piglets, whether it confers greater benefits in low birth weight piglets, and whether synergistic effects exist among the three MO monomers. Therefore, the present study was conducted to investigate the effects of oral administration of this compound milk oligosaccharide (3′-SL:2′-FL:6′-SL = 5:4:1) on growth performance, immune function, and intestinal health in normal and low birth weight suckling piglets, as well as the underlying mechanisms. The findings will enhance our understanding of the functional roles of milk oligosaccharides in suckling piglets and provide experimental evidence to support the inclusion of milk oligosaccharides in milk replacers designed for offspring from high-prolific sows.

## 2. Materials and Methods

### 2.1. Materials

3′-SL, 2′-FL, and 6′-SL, with a purity ≥ 90%, were provided by the Institute of Process Engineering, Chinese Academy of Sciences (Beijing, China). Prior to the experiment, the three milk oligosaccharides were mixed in a mass ratio of 3′-SL:2′-FL:6′-SL = 5:4:1 and dissolved in sterile physiological saline to a final concentration of 37.5 mg/mL (*w*/*v*) to obtain the CMO solution.

### 2.2. Animals, Experimental Design, and Diet

Ten lactating sows with identical parity and litter size were used in this experiment. Four neonatal piglets were selected from each litter, including two normal birth weight (NBW) and two low birth weight (LBW) piglets. Piglets with birth weight < 1.0 kg were classified as LBW, and those with birth weight ≥ 1.0 kg were classified as NBW [40,41]. The experiment was arranged as a 2 × 2 factorial design within a randomized complete block design, with sow litter serving as the block. Two birth weight levels (NBW and LBW) and two CMO supplementation levels (0 mg/d and 75 mg/d) were established to form four treatment groups, with 10 replicates per treatment and one piglet per replicate. The 75 mg/d CMO dose was determined based on our unpublished dose-gradient preliminary trial in neonatal piglets. Gradient doses of 0, 25, 50, 75 and 100 mg/d were tested in the preliminary trial, and the 75 mg/d dose improved growth performance, intestinal morphology and immune function without adverse reactions. Stratified randomization was performed by birth weight within each litter block: the two NBW piglets and two LBW piglets in each litter were separately and randomly assigned to the two CMO treatments. All piglets received daily oral gavage of 2 mL CMO solution or an equal volume of saline from birth to 21 days of age. No creep feed was provided throughout the entire experimental period.

All sows were fed the same lactation diet, with nutrient levels formulated according to NRC (2012) recommendations [42]. As shown in Table 1.

### 2.3. Pig Husbandry

The experiment was conducted in a commercial pig farm with a sow herd of 2400 head. Prior to the trial, the farrowing rooms were thoroughly washed and disinfected, followed by fumigation with potassium permanganate and formaldehyde at a ratio of 1:2. During the experiment, the feeding management (feeding time, frequency, etc.) and vaccination protocols for the sows followed the farm’s standard operating procedures and were kept consistent across all treatments. Each piglet was orally gavaged with 2 mL compound milk oligosaccharide solution using a gavage needle at a fixed time of 12:00–13:00 every day, and this procedure did not interfere with the normal suckling of piglets.

### 2.4. Sample Collection

At the end of the experiment, all piglets were individually weighed. Subsequently, from each treatment group, eight piglets with body weight close to the group mean were selected for blood collection via the anterior vena cava. 10 mL of blood was drawn into vacuum tubes. After standing at room temperature for 30 min, the blood samples were centrifuged at 1780× *g* for 15 min to separate the serum, which was aliquoted into Eppendorf tubes and stored at −20 °C until analysis.

After blood collection, the piglets were slaughtered and dissected. A segment of approximately 2 cm from the middle jejunum was excised, gently rinsed with pre-cooled saline to remove contents, and immediately fixed in 4% paraformaldehyde for histomorphological analysis. In addition, a section of the ileum was opened longitudinally, rinsed with saline to remove luminal contents, blotted dry with filter paper, and the ileal mucosa was gently scraped using a glass slide on ice. The mucosal samples were immediately frozen in liquid nitrogen, and stored at −80 °C until analysis.

After slaughter, the abdominal cavity was opened and the colon was isolated. Colonic digesta were collected and immediately frozen in liquid nitrogen, and then stored at −80 °C for further analysis.

### 2.5. Growth Performance

All piglets were weighed individually on day 0, 7, 14, and 21 of age to calculate the average daily gain (ADG).

### 2.6. Intestinal Morphology

Jejunal samples fixed in 4% paraformaldehyde were dehydrated through a graded ethanol series, cleared in xylene, and embedded in paraffin. Sections of 2 µm thickness were stained with haematoxylin and eosin. Observations were made under a Motic BA210 biological microscope (Motic China Group Co., Ltd., Xiamen, China) at 40× magnification, and ten well-oriented villi (with intact villus structure) were selected and photographed for each sample. Villus height and crypt depth were measured, and the ratio of villus height to crypt depth (V/C) was calculated.

### 2.7. Serum Immune Parameters

The concentrations of serum cytokines (IL-1β, IL-6, IL-10, and TNF-α) and immunoglobulins (IgG and IgM) were determined using commercial porcine-specific ELISA kits (Shanghai Enzyme-linked Biotechnology Co., Ltd., Shanghai, China). All procedures were performed strictly according to the manufacturer’s instructions.

### 2.8. Gene Expression of Intestinal Inflammatory Cytokines

Frozen ileal mucosal samples (approximately 0.1 g) were ground under liquid nitrogen and homogenized in 1 mL of RNAiso Plus (TaKaRa Biotechnology Co., Ltd., Dalian, China) for total RNA extraction, following the manufacturer’s protocol. The purity and concentration of total RNA were assessed using a NanoDrop 2000 spectrophotometer (Thermo Fisher Scientific, Waltham, MA, USA). Samples with an OD_260_/OD_280_ ratio between 1.8 and 2.0 were considered acceptable. Subsequently, 1 μg of total RNA from each ileal sample was reverse-transcribed into cDNA using the PrimeScript™ RT Reagent Kit (TaKaRa Biotechnology Co., Ltd., Dalian, China) with gDNA Eraser (TaKaRa Biotechnology Co., Ltd., Dalian, China) according to the manufacturer’s instructions. The reverse transcription protocol consisted of two steps: 37 °C for 15 min, followed by 85 °C for 5 s.

The mRNA expression levels of genes related to intestinal inflammatory responses (*TNF-α*, *IL-1β*, *IL-6*, *IL-10*, *TLR4*, *MyD88*, and *NF-κB*) were measured by quantitative real-time PCR (qPCR) using the primer sequences listed in Table 2. The qPCR reactions were performed using SYBR^®^ Premix Ex Taq™ II (Tli RNaseH Plus) (TaKaRa Biotechnology Co., Ltd., Dalian, China). Each 10 μL reaction mixture contained 5 μL of SYBR Premix Ex Taq II, 0.5 μL of forward primer, 0.5 μL of reverse primer, 1 μL of cDNA, and 3 μL of RNase-free water. The qPCR protocol was: pre-denaturation at 95 °C for 30 s, followed by 40 cycles of 95 °C for 5 s and 60 °C for 34 s. After each run, a melting curve analysis was performed to verify the specificity of the amplified products. The housekeeping gene β-actin was used as an internal reference, and the relative mRNA expression of target genes was calculated using the 2^−ΔΔCt^ method [43].

### 2.9. Colonic Microbiota

Colonic digesta samples from five piglets randomly selected from each treatment group were subjected to 16S rRNA sequencing analysis to characterize the gut microbial community composition. Microbial genomic DNA was extracted from 0.25 g of thawed colonic digesta using the Zymo Research BIOMICS DNA Microprep Kit (Zymo Research, Irvine, CA, USA) according to the manufacturer’s instructions. The integrity of the extracted genomic DNA was assessed by 0.8% (*w*/*v*) agarose gel electrophoresis. Sequencing and bioinformatics analysis were performed by Chengdu Loning Biotechnology Co., Ltd. (Chengdu, China). Paired-end sequencing was carried out on the Illumina HiSeq platform with a small-fragment library constructed for sequencing. High-quality sequences were clustered using QIIME software (version 2023.2) at a 97% similarity threshold to obtain operational taxonomic units (OTUs). The representative sequences of each OTU were aligned against the microbial reference databases to obtain taxonomic information, and the community composition of each sample was subsequently summarized at both the phylum and genus levels. Alpha diversity indices, including the Observed species, ACE, Chao1, Shannon, and Simpson indices, were calculated. For stacked bar charts at the phylum and genus levels, the top 20 phyla and genera with the highest relative abundance were visualized individually. The remaining low abundance taxa and unassigned OTUs were summed and designated as Others. Calculations of relative abundance and Others were performed on each individual sample, before averaging across replicates within each treatment group for figure plotting.

### 2.10. Statistical Analysis

All data were analyzed using the general linear model (GLM) procedure in SPSS 27.0 software for normally distributed datasets. A two-way analysis of variance (two-way ANOVA) was performed with birth weight and CMO treatment as the two fixed factors to evaluate growth performance, intestinal development, serum immune parameters, and intestinal inflammatory cytokine gene expression in suckling piglets. Multiple comparisons among treatment groups were performed using Duncan’s multiple range test for parameters analyzed by two-way ANOVA. For colonic microbiota data, including alpha-diversity indices and the relative abundances of taxa at the phylum and genus levels, the Scheirer-Ray-Hare non-parametric factorial test was implemented in R (version 4.3.2) using the rcompanion package. When a significant interaction was observed (*p* < 0.05), post hoc multiple comparisons were conducted using Dunn’s test with Benjamini–Hochberg *p*-value correction via the *FSA* package (version 0.9.5). All data are presented as means and standard error of the mean (SEM). A probability value of *p* < 0.05 was considered statistically significant, while 0.05 ≤ *p* < 0.10 was regarded as indicating a tendency. Graphical illustrations were generated using GraphPad Prism 10.0 software. The sample size was based on our preliminary dose-gradient trial and previous piglet studies. Post hoc power analysis using G*Power 3.1 (2 × 2 factorial ANOVA, α = 0.05, *n* = 5 per group) indicated power > 0.80 for large phylum/genus-level effects (e.g., Synergistetes), while power for alpha-diversity interaction effects was approximately 0.60–0.65. Therefore, the microbiota results are interpreted as exploratory and hypothesis-generating.

## 3. Results

### 3.1. Effects of Compound Milk Oligosaccharides on Growth Performance of Suckling Piglets

As shown in Table 3, normal birth weight piglets had significantly higher body weights than that of low birth weight piglets throughout the whole experimental period (*p* < 0.05). Oral administration of CMO solution significantly increased body weight on days 14 and 21, as well as average daily gain (ADG) during days 0–14 and 0–21, in both normal and low birth weight piglets (*p* < 0.05). No significant interaction effects between birth weight and CMO supplementation were observed on body weight or ADG of suckling piglets during the trial (*p* > 0.05).

### 3.2. Effects of Compound Milk Oligosaccharides on Intestinal Morphology of Suckling Piglets

**Figure 1:**
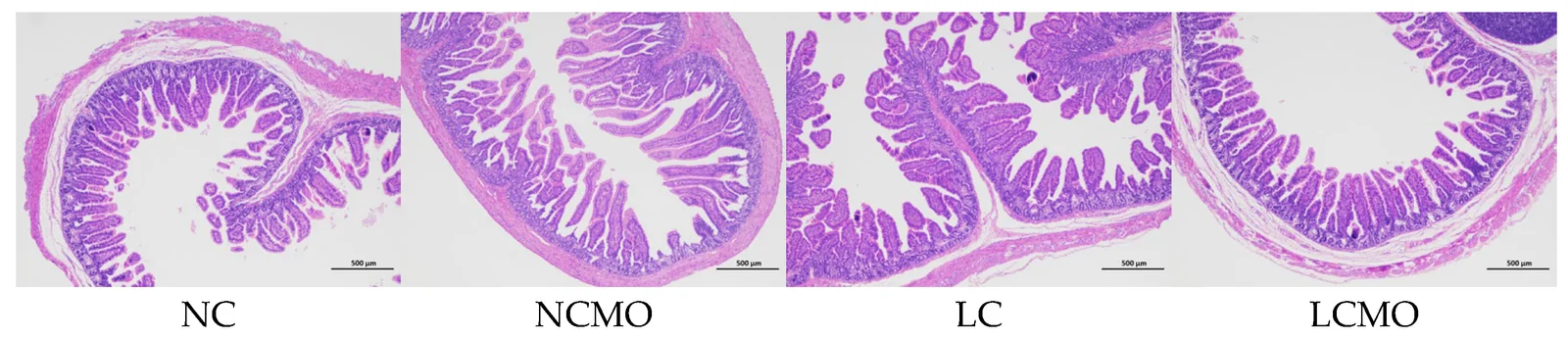
Effects of CMO on jejunal morphology of suckling piglets with different birth weights (Hematoxylin and eosin staining, ×40). NC: normal birth weight piglets orally administered 2 mL of 0.9% saline; NCMO: normal birth weight piglets orally administered 2 mL of CMO solution (37.5 mg/mL); LC: low birth weight piglets orally administered 2 mL of 0.9% saline; LCMO: low birth weight piglets orally administered 2 mL of CMO solution (37.5 mg/mL). The same as below.

As shown in Table 4 and Figure 1, at the end of the experiment, normal birth weight piglets had significantly greater jejunal villus height and the ratio of villus height-to-crypt depth (V/C) than that of low birth weight piglets (*p* < 0.05). Oral administration of CMO solution significantly increased jejunal villus height and V/C ratio in both normal and low birth weight piglets (*p* < 0.05). No significant interaction effects between birth weight and CMO supplementation were observed on jejunal villus height, crypt depth, or V/C ratio at the end of the trial (*p* > 0.05).

### 3.3. Effects of Compound Milk Oligosaccharides on Serum Immune Parameters of Suckling Piglets

As shown in Table 5, at the end of the experiment, the serum concentrations of TNF-α, IL-1β, IL-6, and IL-10 in normal birth weight piglets were significantly higher than those in low birth weight piglets (*p* < 0.05). Oral administration of CMO solution significantly reduced serum IL-1β, IL-6, and IL-10 concentrations in both normal and low birth weight piglets (*p* < 0.05). An interaction between birth weight and CMO supplementation was observed, tending to reduce serum IL-6 concentration at the end of the experiment (*p* = 0.093). In normal birth weight piglets, CMO supplementation was associated with a 48.71% decrease in IL-6 concentration compared with the control group.

### 3.4. Effects of Compound Milk Oligosaccharides on Inflammatory Cytokines and TLR4 Signaling Pathway in the Ileum of Suckling Piglets

**Figure 2:**
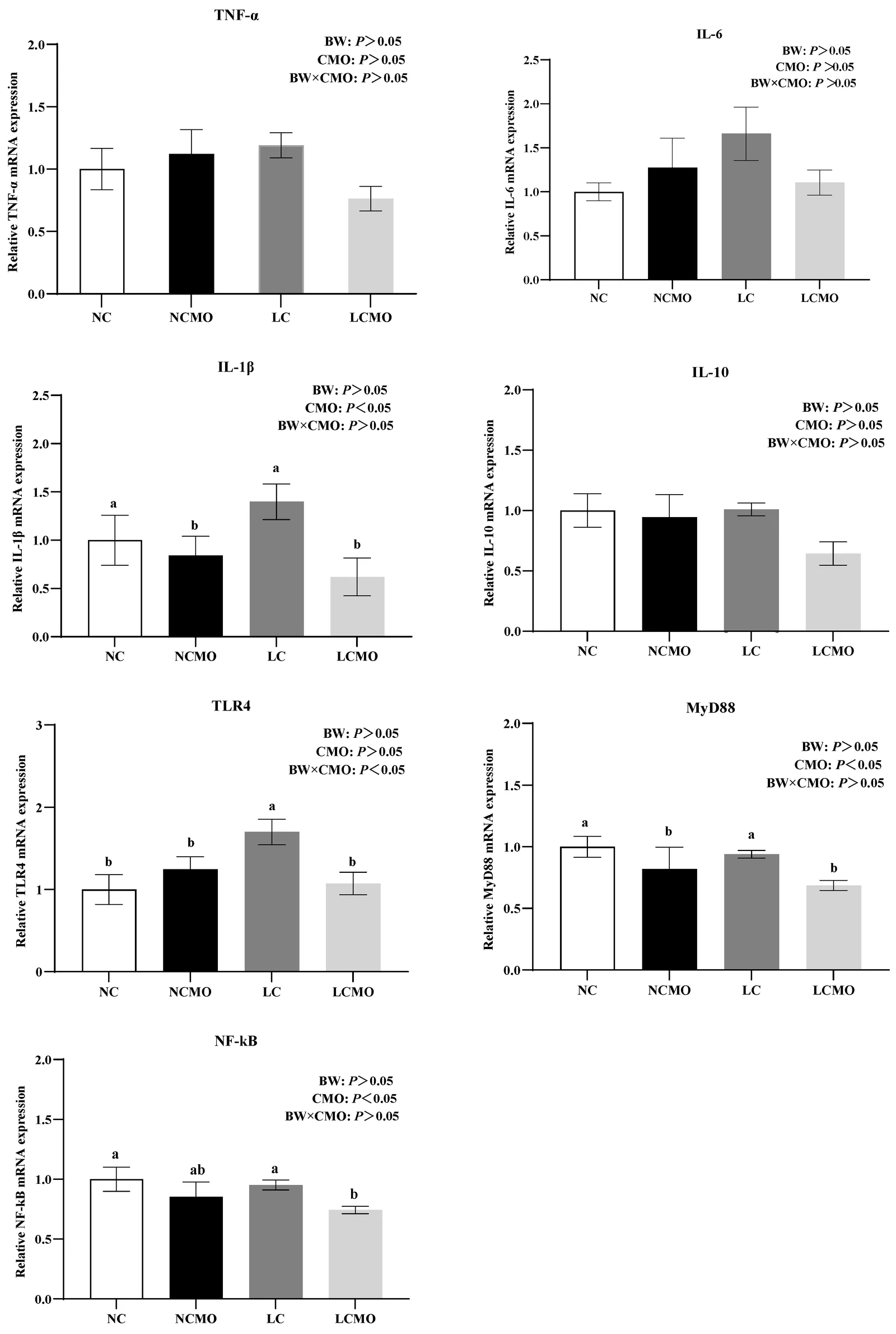
Effects of CMO on inflammatory cytokines and TLR4 signaling pathway in the ileum of suckling piglets with different birth weights. Different lowercase letters (a, b, ab) above the bars indicate significant differences among groups (*p* < 0.05); values sharing the same letter or a combination of letters are not significantly different.

As shown in Figure 2, birth weight of piglets had no significant effect on the gene expression of inflammatory cytokines and the TLR4 signaling pathway in the ileum (*p* > 0.05). Oral administration of CMO solution significantly decreased the ileal mRNA expression of *IL-1β* and *MyD88* in both normal and low birth weight piglets, as well as *NF-κB* expression in low birth weight piglets (*p* < 0.05). There were no significant interaction effects between birth weight and CMO supplementation on the ileal gene expression of *TNF-α*, *IL-1β*, *IL-6*, *IL-10*, *MyD88*, and *NF-κB* at the end of the experiment (*p* > 0.05). However, a significant interaction effect was observed on ileal *TLR4* gene expression (*p* < 0.05), evidenced by a significant reduction in *TLR4* expression in low birth weight piglets supplemented with CMO (*p* < 0.05).

### 3.5. Effects of Compound Milk Oligosaccharides on Colonic Digesta Microbiota of Suckling Piglets

As shown in Table 6, piglet birth weight and CMO had no significant effect on the diversity and abundance of colonic digesta microbiota in suckling piglets; however, significant interaction effects between birth weight and CMO supplementation on observed species, Chao 1 and ACE were observed. Oral administration of CMO significantly increased observed species, Chao 1 and ACE in normal birth weight piglets’ colonic digesta microbiota, while decreasing these parameters in low birth weight piglets (*p* < 0.05).

**Figure 3:**
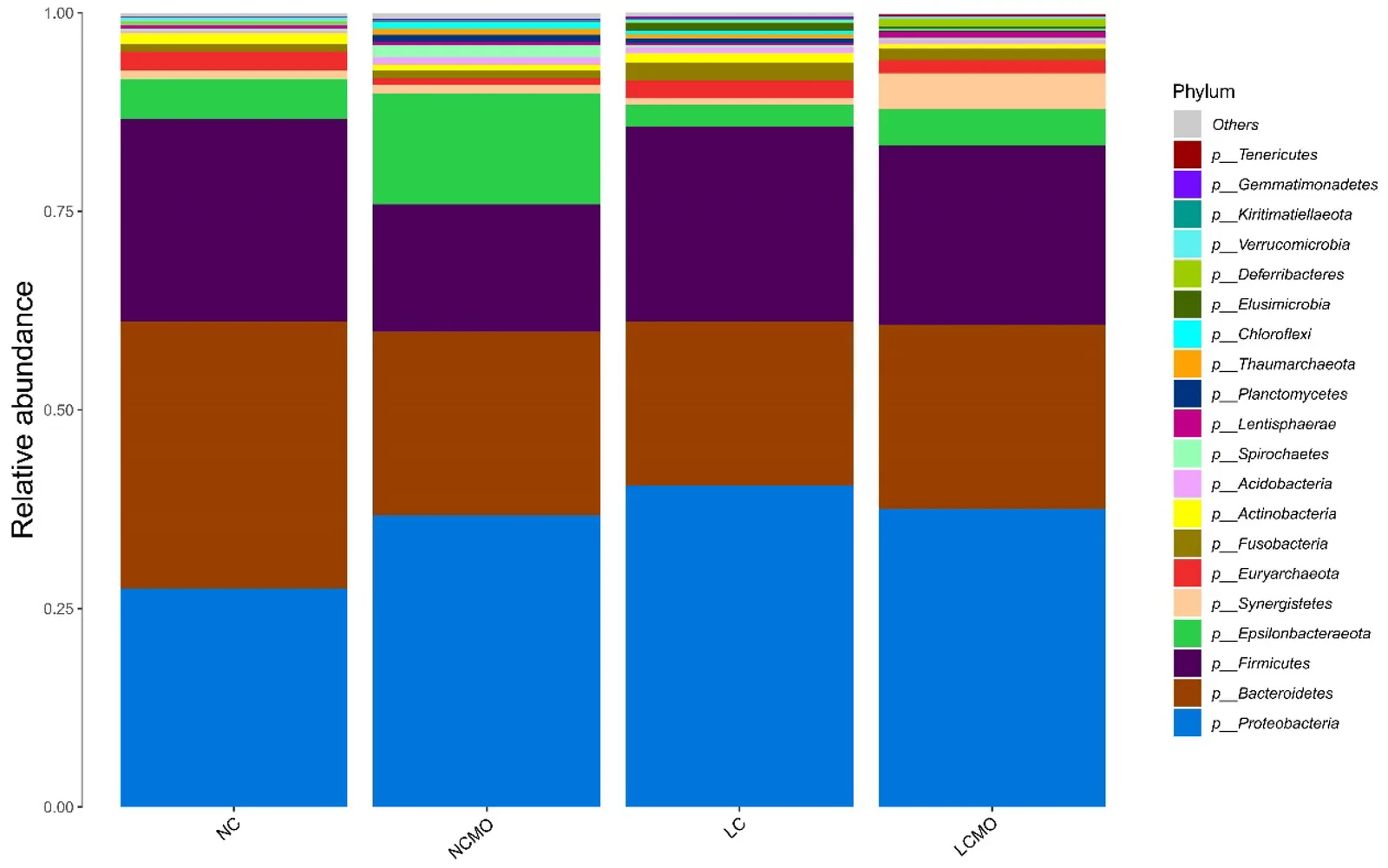
Effects of CMO on the phylum-level distribution of colonic microbiota in suckling piglets (top 20 in relative abundance).

**Figure 4:**
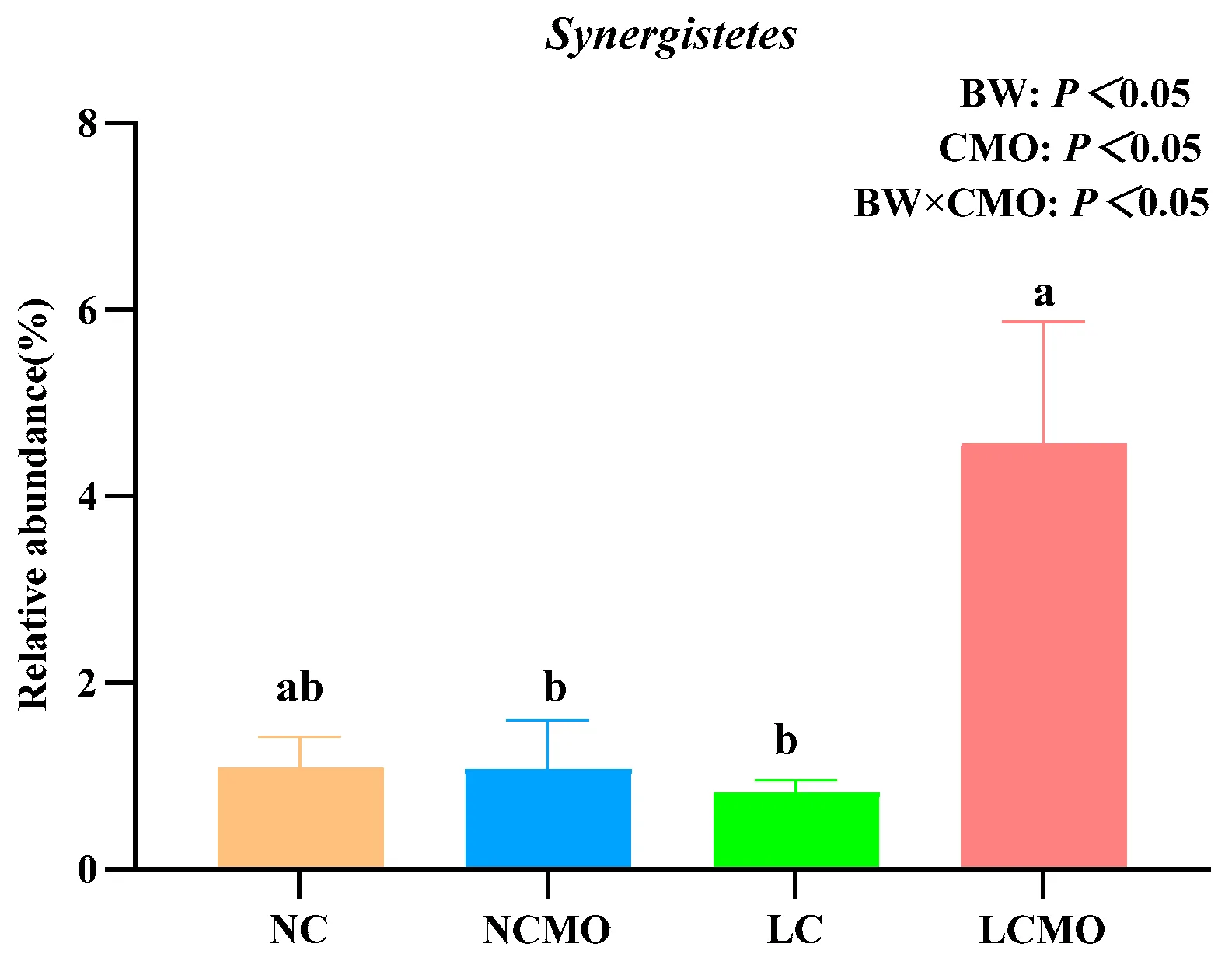
Differential microorganisms at the phylum level. Different lowercase letters (a, b, ab) above the bars indicate significant differences among groups (*p* < 0.05); values sharing the same letter or a combination of letters are not significantly different.

As shown in Figure 3, the top 20 phyla with the highest relative abundance were detected in all samples. At the phylum level, *Proteobacteria*, *Bacteroidota*, and *Firmicutes* were the dominant phyla in the colonic microbiota of suckling piglets, accounting for more than 86% of all phyla. The stacked bar plot revealed a numerically higher relative abundance of *Proteobacteria* in low birth weight piglets compared with normal birth weight piglets. As shown in Figure 4, there was a significant interaction effect between piglet birth weight and CMO supplementation on the relative abundance of *Synergistetes* in the colon (*p* < 0.05). Specifically, the relative abundance of *Synergistetes* in the colon of low birth weight piglets supplemented with CMO was significantly higher than that in normal birth weight piglets supplemented with CMO (*p* < 0.05).

**Figure 5:**
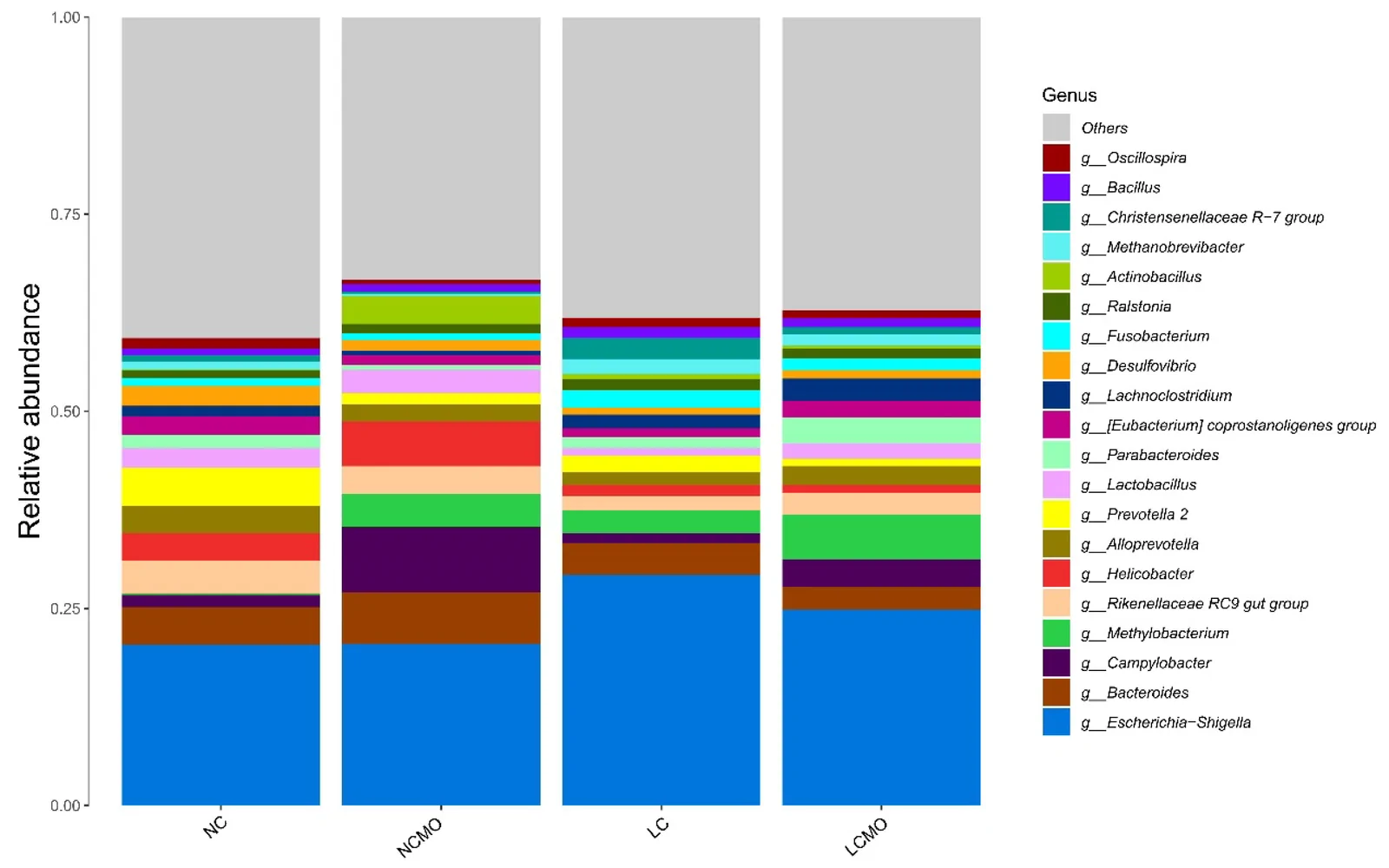
Effects of CMO on the genus-level distribution of colonic microbiota in suckling piglets (top 20 in relative abundance).

**Figure 6:**
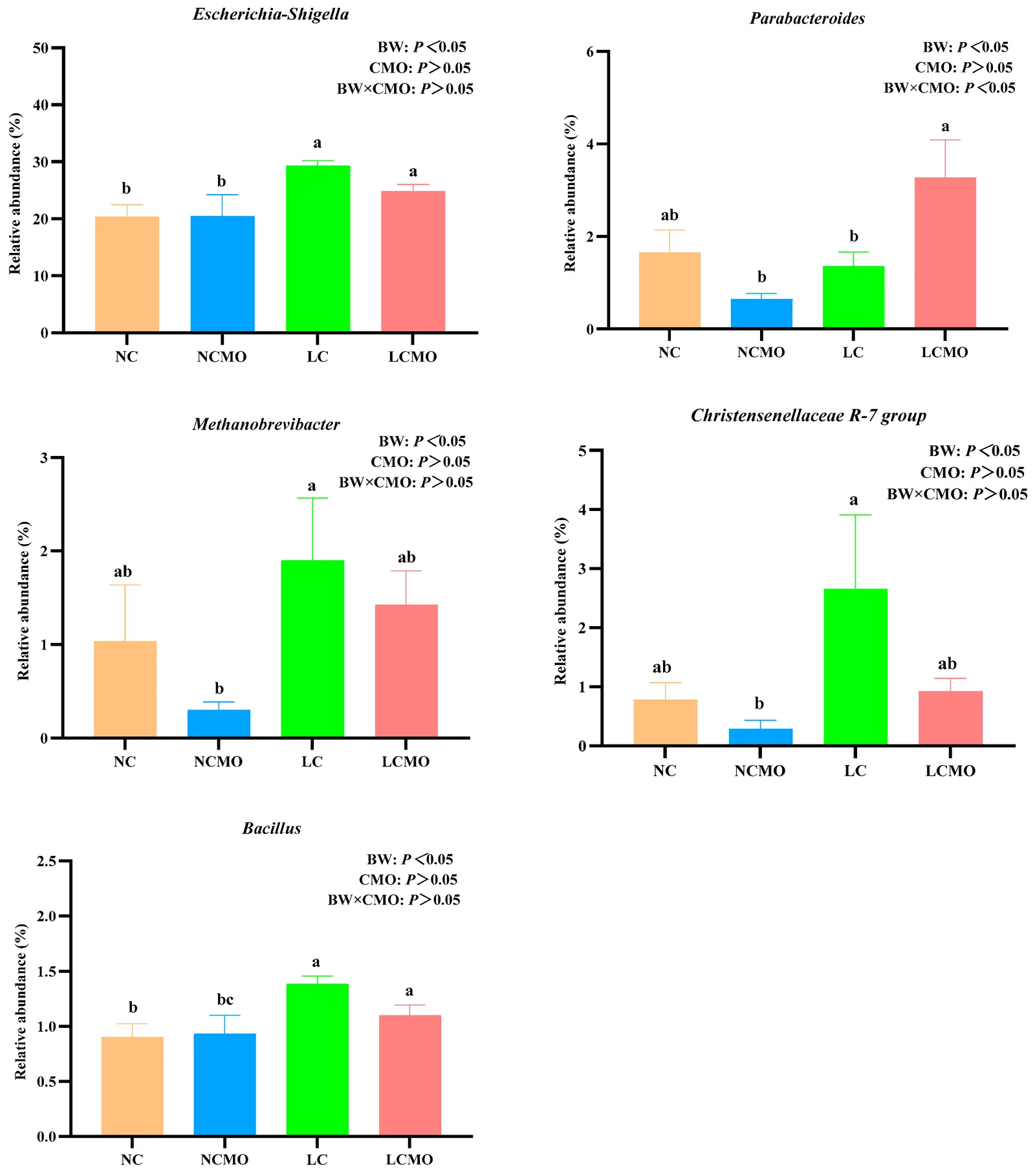
Differential microorganisms at the genus level. Different lowercase letters (a, b, ab, bc) above the bars indicate significant differences among groups (*p* < 0.05); values sharing the same letter or a combination of letters are not significantly different.

Figure 5 shows the top 20 bacterial genera sorted by relative abundance, with low abundance taxa grouped as Others. *Escherichia-Shigella* was the predominant genus in the colon of suckling piglets across all four groups, followed by *Bacteroides*, *Campylobacter*, *Methylobacterium*, *Rikenellaceae* RC9 gut group, and *Lactobacillus*. The stacked bar plot revealed numerically higher relative abundance of *Escherichia-Shigella* in low birth weight groups (LC and LCMO) compared with normal birth weight groups (NC and NCMO). A higher proportion of *Campylobacter* was visually observed in the NCMO group, and the relative abundances of dominant genera fluctuated among different combinations of birth weight and CMO supplementation. As shown in Figure 6, the relative abundance of *Escherichia-Shigella* in the colon of normal birth weight piglets was significantly lower than that of low birth weight piglets (*p* < 0.05), while neither the administration of CMO nor the interaction between birth weight and CMO had any significant effect (*p* > 0.05). For *Parabacteroides*, both piglet birth weight (*p* < 0.05) and its interaction with CMO supplementation (*p* < 0.05) significantly affected its relative abundance. Specifically, the relative abundance of *Parabacteroides* in the colon of low birth weight piglets receiving CMO solution was significantly higher than that in normal birth weight piglets receiving the same CMO treatment (*p* < 0.05, and within the low birth weight group, CMO supplementation significantly increased its relative abundance (*p* < 0.05). Birth weight also significantly influenced the relative abundances of *Methanobrevibacter* and the *Christensenellaceae* R-7 group (*p* < 0.05), with low birth weight piglets showing significantly higher relative abundances of these two genera compared with normal birth weight piglets supplemented with CMO (*p* < 0.05). Furthermore, the relative abundance of *Bacillus* was significantly higher in low birth weight piglets than in normal birth weight piglets (*p* < 0.05); however, neither CMO supplementation nor the interaction between birth weight and CMO exerted a significant effect (*p* > 0.05).

## 4. Discussion

MOs are abundant and structurally complex components of mammalian milk, possessing multiple biological activities. However, in current commercial production systems, the increase in litter size of high-prolific sows is not accompanied by a proportional increase in total milk yield, leading to a reduced per-piglet intake of naturally occurring CMOs via sow’s milk. Consequently, exogenous supplementation of MOs during the lactation period may represent a promising direction for the development of efficient milk replacers for piglets. Nevertheless, studies investigating the effects of such supplementation on the health and growth of suckling piglets remain limited.

Previous studies have shown that dietary sialyllactose supplementation improved average daily gain and feed efficiency in weaned piglets challenged with enterotoxigenic *Escherichia coli* (ETEC) [32]. Oral administration of sialyllactose to suckling piglets significantly increased weaning litter weight [25]. In a neonatal piglet model colonized with gut microbiota from malnourished Malawian infants, sialyllactose was found to enhance growth performance and improve nutrient utilization through remodeling of the fecal microbiota [44]. Kim et al. reported that feeding a moderate-dose MOs mixture (2′ -fucosyllactose and sialyllactose) to suckling mice for 2 weeks alleviated body weight loss induced by LPS challenge [38]. Furthermore, to our knowledge, only one study has reported that oral administration of sialyllactose to suckling piglets significantly increased litter weaning weight [25]. Therefore, based on our previous work on the individual effects of 3′-SL, 2′-FL, and 6′-SL in suckling piglets (data unpublished), and taking into account the relative proportions of these three oligosaccharides in porcine milk, we formulated a compound milk oligosaccharide (CMO) mixture at a mass ratio of 5:4:1 (3′-SL:2′-FL:6′-SL). The results of the present study showed that oral administration of this CMO solution during the suckling period significantly increased average daily gain in both normal birth weight and low birth weight piglets over days 0–14 and 0–21, indicating that CMO effectively improved growth performance in suckling piglets.

The intestinal barrier is an important structural foundation for intestinal function. Studies have shown that sialyllactose can maintain intestinal barrier integrity by improving intestinal morphology and enhancing the expression of tight junction proteins [32]. Small intestinal villi are essential components of the digestive tract, and their health status is routinely evaluated by villus height, crypt depth, and the villus height-to-crypt depth ratio (VH/CD) [45]. Li et al. [25] reported that oral administration of sialyllactose to suckling piglets significantly increased jejunal villus height and the VH/CD ratio, and the underlying mechanism was closely associated with enhanced immune function, improved antioxidant capacity, and strengthened intestinal barrier integrity. Furthermore, Duan et al. [32] found that ETEC challenge significantly reduced jejunal and the VH/CD ratio in both the jejunum and ileum of weaned piglets, while dietary sialyllactose supplementation significantly increased these parameters in both challenged and non-challenged piglets. In the present study, oral administration of CMO solution significantly increased jejunal villus height and the VH/CD ratio in both normal and low birth weight suckling piglets. Collectively, these findings indicate that MOs can promote intestinal development in piglets.

Immunoglobulins are glycoprotein molecules produced by plasma cells that participate in immune responses by specifically recognizing and binding to particular antigens (e.g., bacteria or viruses), thereby neutralizing their pathogenic effects [46]. Li et al. [25] reported that oral administration of sialyllactose to suckling piglets significantly increased serum IgG concentration, thereby enhancing systemic immunity. However, the present study found that oral administration of CMO solution did not significantly affect serum IgG and IgM concentrations in either normal or low birth weight piglets, which differs from previous findings. This discrepancy may be attributable to differences in the health status of animals and dosages administered. Pro-inflammatory cytokines such as TNF-α, IL-1β, and IL-6 can regulate immune status and facilitate rapid host immune responses following pathogen infection [47]. Castillo et al. demonstrated that oral administration of 6′-SL or 2′-FL in mice increased the population of CD4^+^CD25^+^IL-10^+^ regulatory T cells, which suppress excessive immune responses through secretion of the anti-inflammatory cytokine IL-10. Moreover, 6′-SL promoted IL-10 secretion and inhibited TNF production, thereby alleviating food allergy symptoms in mice [48]. In this study, lower basal serum IL-1β and IL-6 levels in LBW piglets indicate immune immaturity rather than superior health status. Oral CMO administration significantly decreased serum IL-1β, IL-6, and IL-10 concentrations in suckling piglets, suggesting that CMO attenuated systemic pro-inflammatory responses. However, because IL-10 is an anti-inflammatory cytokine, the concurrent reduction in IL-10 should be interpreted with caution. It may reflect a negative feedback mechanism after inflammation is alleviated, but could also indicate altered or immature immune regulation in LBW piglets. Therefore, the lower IL-10 concentration does not necessarily represent an anti-inflammatory shift. These findings demonstrate that CMO modulates the immune system of suckling piglets from a hyper-responsive “alert” state toward a more mature and stable immune homeostasis.

Toll-like receptors (TLRs) are prototypical antigen-recognition receptors that participate in inflammatory responses by mediating innate defense of the intestinal mucosa and activating the TLR4-MyD88-NF-κB signaling pathway [49,50]. Blockade of the MyD88 signaling pathway (an upstream activator of the NF-κB pathway) inhibits *NF-κB* activation, thereby reducing the release of pro-inflammatory cytokines and alleviating inflammatory responses [51]. In the present study, oral administration of CMO during the suckling period reduced ileal mucosal mRNA expression of *IL-1β* and *MyD88* in both NBW and LBW piglets, whereas *NF-κB* expression was significantly reduced only in LBW piglets. These changes were partially consistent with the observed reductions in serum IL-1β, IL-6, and IL-10 concentrations. These findings suggest that CMO may attenuate the TLR4/MyD88/NF-κB signaling pathway at the mRNA level, with more pronounced regulation of *TLR4* and *NF-κB* in LBW piglets, thereby helping to restore inflammatory homeostasis. Previous studies have reported that 2′-FL and 6′-SL can prevent necrotizing enterocolitis in mouse and piglet models by suppressing TLR4 signaling [39]. Similarly, Duan et al. reported that dietary sialyllactose reduced the expression of intestinal mucosal inflammatory cytokines (*TNF-α*, *IL-1β*, and *IL-6*) and key molecules of the inflammatory signaling pathway (MyD88 and NF-κB) in weaned piglets challenged with ETEC [52]. However, in our study, a significant interaction between birth weight and CMO supplementation was observed on *TLR4* gene expression, CMO significantly decreased ileal *TLR4* expression in low birth weight piglets, whereas the interaction for serum IL-6 was only a marginal trend, with a numerically greater reduction in normal birth weight piglets. We hypothesize that in normal birth weight piglets, which possess a more mature immune system, CMO primarily exerts a systemic regulatory effect; in contrast, in low birth weight piglets, CMO appears to act preferentially as an intestinal restorative agent, potently downregulating intestinal *TLR4* (with a significant interaction) to attenuate the source of inflammation; however, the underlying mechanisms need further investigation.

The gut microbiota plays an important role in maintaining intestinal health. It has been well established that MOs can modulate host intestinal homeostasis by altering the composition of the gut microbial community [25,34]. In line with previous reports [50,53,54], the phyla Firmicutes and Bacteroidetes predominated in the colonic microbiota of this experimental piglets, which is consistent with the general consensus. The phylum Synergistetes is recognized as a short-chain fatty acid (SCFA)-producing group, with acetate, propionate, and formate as their main metabolites [55]. SCFAs have been shown to alleviate intestinal inflammation in piglets [56]. Therefore, an increased abundance of Synergistetes may contribute to the alleviation of intestinal inflammatory responses. In this study, a significant interaction effect between birth weight and CMO supplementation was observed on the relative abundance of Synergistetes: oral administration of CMO significantly increased the abundance of this phylum in low birth weight piglets. This suggests that CMO supplementation is particularly beneficial for improving the hindgut microbial balance in low birth weight piglets. Moreover, this finding was inversely correlated with the effects of CMO on ileal *TLR4*, *MyD88*, *NF-κB*, and *IL-1β* gene expression as well as on serum IL-1β, IL-6, and IL-10 concentrations, implying that CMO may exert its anti-inflammatory effects, at least in part, through modulation of the hindgut microbiota.

At the genus level, the relative abundance of *Escherichia-Shigella* was significantly lower in normal birth weight piglets than in low birth weight piglets. Some members of this genus are known enteropathogens in pigs [57], and previous studies have reported a higher abundance of *Escherichia-Shigella* in the jejunum of intrauterine growth-restricted (IUGR) piglets [58]. However, other investigations have found no significant difference in *Escherichia-Shigella* abundance between very low birth weight and normal birth weight infants [59], which may be attributable to differences in species and age. In contrast, *Parabacteroides* is considered a beneficial genus that can modulate the mucosal immune system and alleviating inflammation by producing acetate and reducing neutrophil infiltration [60,61]. In the present study, CMO administration significantly increased the relative abundance of *Parabacteroides* in low birth weight piglets. This is consistent with a previous study in a colitis mouse model, where 2′-FL intervention significantly increased the relative abundance of *Bacteroidetes* and *Parabacteroides* [62]. Additionally, the genus *Methanobrevibacter* has been reported to be highly enriched in the hindgut of growth-retarded piglets, where it primarily inhibits hepatic ketone body metabolism, leading to growth suppression [63]. In agreement with that finding, in this study, the relative abundance of *Methanobrevibacter* in the colon of low birth weight piglets was significantly higher than that of normal birth weight piglets supplemented with CMO. *Bacillus* and the *Christensenellaceae* R-7 group have been demonstrated to be associated with intestinal health, by improving intestinal barrier function and modulating microbial balance [64,65]. Interestingly, in the present study, the relative abundances of *Bacillus* and the *Christensenellaceae* R-7 group were significantly higher in CMO-supplemented low birth weight piglets than in CMO-supplemented normal birth weight piglets. For this unexpected finding, since microbiota data are compositional, the difference may represent either a true increase in these taxa or a relative abundance shift resulting from changes in other community members, such as the reduction in pathogenic taxa including *Escherichia-Shigella*. However, absolute quantification (qPCR) was not performed in this study; therefore, we cannot distinguish between these two possibilities. This only demonstrates a change in relative abundance and cannot directly prove the proliferation of bacterial absolute quantity, and further validation is required.

Moreover, a major limitation of this study is that CMO was administered at a fixed oral dose (75 mg/day) rather than a body-weight-adjusted dose. Because LBW piglets had approximately half the body weight of NBW piglets at birth, the body-weight-normalized CMO exposure on day 0 was approximately 97 mg/kg in LBW piglets versus 48 mg/kg in NBW piglets. Therefore, the more pronounced anti-inflammatory and microbiota-modulating effects observed in LBW piglets, including the downregulation of ileal *TLR4* and *NF-κB* and the enrichment of *Parabacteroides* and *Synergistetes*, may partly reflect higher relative CMO exposure rather than an intrinsic birth weight-dependent response. Thus, future studies using body-weight-adjusted dosing or multiple dose levels are needed to disentangle the effects of absolute CMO intake from those of birth weight.

## 5. Conclusions

In summary, our findings demonstrate that a daily oral dose of 75 mg CMO consistently improves growth performance and gut morphology of suckling piglets across birth weight, yet its anti-inflammatory and microbiota-modulating effects are partly birth weight-dependent. The underlying mechanisms may be closely associated with the improvement in intestinal integrity and the attenuation of TLR4/MyD88/NF-κB signaling, and modulation of gut microbial balance. These effects collectively improved overall growth performance.

## Figures and Tables

**Table 1:** Composition and nutrient levels of the basal diet for sow (air-dry basis).

Ingredients	%
Corn	32.12
Wheat meal, 14% CP	30.00
Wheat bran	10.11
Soybean meal, 43% CP	8.34
Expanded soybean	5.00
Fermented soybean meal	5.00
Sugar beet meal granulate	2.00
Soybean oil	2.00
Expanded flaxseed	2.00
Calcium hydrogen phosphate type I	1.32
Limestone	0.80
Sodium chloride, 50%	0.40
*L*-Lys HCL, 98%	0.33
*L*-Thr, 98%	0.13
*DL*-Met, 98.5%	0.03
*L*-Trp, 98%	0.02
*L*-Val, 98.5%	0.05
Choline chloride, 50%	0.10
Mineral premix ^1^	0.20
Vitamin premix ^2^	0.05
Total	100.00
Nutrient level	
Digestible energy, Mcal/kg	3.38
Crude protein, %	17.00
Calcium, %	0.80
Total phosphorus, %	0.62
SID Lys	0.90
SID Methionine + Cysteine	0.50
SID Thr	0.59
SID Trp	0.22
SID Val	0.72

^1^ The mineral premix provided the following per kg of diet: Cu (CuSO_4_.5H_2_O), 20 mg; Fe (FeSO_4_.H_2_O), 150 mg; Zn (ZnSO_4_.H_2_O), 100 mg; Mn (MnSO_4_.H_2_O), 50 mg; I (Ca(IO_3_)_2_), 1 mg; Se (Na_2_SeO_3_), 0.25 mg. ^2^ The vitamin premix provided the following per kg of diet:: VA, 7000 IU; VD_3_, 2100 IU; VE, 100 IU; VK_3_, 2.1 mg; VB_1_, 1.25 mg; VB_2_, 7.65 mg; VB_6_, 4.65 mg; VB_12_, 30 μg; biotin, 0.6 mg; folic acid, 3.5 mg; pantothenic acid, 20 mg; nicotinic acid, 35 mg.

**Table 2:** Primer sequences for real-time quantitative PCR.

Gene	Primer Sequence (5′-3′)	Product Size (bp)
*β-actin*	F:TGGAACGGTGAAGGTGACAGC	177
	R:GCTTTTGGGAAGGCAGGGACT	
*TNF-a*	F:AGCTGAACTGTAGGTTGCTCC	178
	R:AGGGCGGTTACAGACACAAC	
*IL-6*	F:TGTCGAGGCTGTGCAGATTAG	80
	R:TGGTGGCTTTGTCTGGATTCT	
*IL-10*	F:TGGAAGACGTAATGCCGAAGG	171
	R:TGCTAAAGGCACTCTTCACCTC	
*IL-1β*	F:CAGCTGCAAATCTCTCACCA	85
	R:TCTTCATCGGCTTCTCCACT	
*NF-kB*	F:CATCTTTGACAACCGTGCCC	147
	R:GGGCCCGTGAAATACACCTC	
*TLR4*	F:TTACAGAAGCTGGTTGCCGT	152
	R:TCCAGGTTGGGCAGGTTAGA	
*MyD88*	F:CCATTCGAGATGACCCCCTG	183
	R:TAGCAATGGACCAGACGCAG	

**Table 3:** Effects of CMO on growth performance of suckling piglets with different birth weights.

Items	Treatments ^1^				SEM	*p*-Value		
	NC	NCMO	LC	LCMO		BW	CMO	BW × CMO
BW, kg								
0 d	1.58 ^a^	1.56 ^a^	0.77 ^b^	0.78 ^b^	0.07	<0.001	0.979	0.505
7 d	2.98 ^a^	3.10 ^a^	1.68 ^b^	1.83 ^b^	0.12	<0.001	0.130	0.908
14 d	5.14 ^b^	5.56 ^a^	3.09 ^c^	3.46 ^b^	0.20	<0.001	0.013	0.876
21 d	7.05 ^b^	7.81 ^a^	4.52 ^c^	5.18 ^b^	0.26	<0.001	0.004	0.826
ADG, g/d								
0–7 d	199.82 ^a^	219.82 ^a^	130.71 ^b^	149.11 ^b^	8.67	<0.001	0.122	0.947
0–14 d	253.93 ^b^	285.27 ^a^	165.89 ^c^	191.61 ^b^	9.88	<0.001	0.011	0.789
0–21 d	260.3 ^b^	297.56 ^a^	177.86 ^c^	209.64 ^b^	9.68	<0.001	0.003	0.798

^1^ NC: Normal birth weight piglets oral administration of 2 mL of 0.9% saline; NCMO: Normal birth weight piglets oral administration of 2 mL of 37.5 mg/mL CMO solution; LC: Low birth weight piglets oral administration of 2 mL of 0.9% saline; LCMO: Low birth weight piglets oral administration of 2 mL of 37.5 mg/mL CMO solution. The same as below. ^a,b,c^ Means with different superscript letters in a row were significantly different (*p* < 0.05). The same as below.

**Table 4:** Effects of CMO on jejunal morphology of suckling piglets with different birth weights.

Items	Treatments				SEM	*p*-Value		
	NC	NCMO	LC	LCMO		BW	CMO	BW × CMO
Villus height, μm	218.56 ^b^	258.5 ^a^	177.29 ^c^	237.45 ^b^	8.47	0.028	0.001	0.456
Crypt depth, μm	125.12	108.50	122.15	127.53	5.81	0.509	0.643	0.368
V:C	1.91 ^b^	2.51 ^a^	1.51 ^c^	2.05 ^b^	0.10	0.008	0.001	0.844

^a,b,c^ Means with different superscript letters in a row were significantly different (*p* < 0.05).

**Table 5:** Effects of CMO on serum immune parameters of suckling piglets with different birth weights.

Items	Treatments				SEM	*p*-Value		
	NC	NCMO	LC	LCMO		BW	CMO	BW × CMO
IgG, mg/mL	1.60	1.41	1.37	1.60	0.09	0.909	0.924	0.238
IgM, mg/mL	0.28	0.15	0.22	0.14	0.03	0.503	0.055	0.573
TNF-α, pg/L	415.38 ^a^	352.69 ^a^	283.80 ^b^	295.32 ^b^	17.92	0.006	0.420	0.246
IL-1β, ng/L	71.82 ^a^	57.31 ^b^	44.55 ^b^	38.46 ^c^	2.96	<0.001	0.005	0.221
IL-6, ng/L	2593.69 ^a^	1344.25 ^b^	1106.19 ^b^	803.17 ^c^	184.84	0.001	0.009	0.093
IL-10, ng/L	445.85 ^a^	336.81 ^b^	202.32 ^b^	180.21 ^c^	25.76	<0.001	0.046	0.174

^a,b,c^ Means with different superscript letters in a row were significantly different (*p* < 0.05). The same as below.

**Table 6:** Effects of CMO on colonic digesta microbial diversity and abundance in suckling piglets with different birth weights.

Items	Treatments				SEM	*p*-Value		
	NC	NCMO	LC	LCMO		BW	CMO	BW × CMO
Observed species	559.40 ^b^	767.00 ^a^	826.40 ^a^	613.00 ^ab^	50.22	0.560	0.976	0.041
Chao1	573.45 ^b^	793.57 ^a^	850.06 ^a^	636.44 ^ab^	51.65	0.550	0.974	0.041
ACE	571.38 ^b^	790.16 ^a^	850.72 ^a^	637.03 ^ab^	51.78	0.528	0.980	0.042
Shannon	4.27	3.94	4.20	4.04	0.08	0.916	0.145	0.596
Simpson	0.94	0.91	0.90	0.92	0.01	0.247	0.575	0.055

^a,b,ab^ Means with different superscript letters in a row were significantly different (*p* < 0.05). The same as below.

## Data Availability

The original contributions presented in this study are included in the article. Further inquiries can be directed to the corresponding author.

## Abbreviations

The following abbreviations are used in this manuscript:

ADG	Average daily gain
BW	Body weight
CMO	Compound milk oligosaccharide
ETEC	Enterotoxigenic *Escherichia coli*
FL	Fucosyllactose
GL	Galactosyllactose
GOS	Galactooligosaccharides
Ig-G	Immunoglobulin G
Ig-M	Immunoglobulin M
IL-1β	Interleukin-1β
IL-6	Interleukin-6
IL-10	Interleukin-10
LNnT	Lacto-N-neotetraose
MOs	Milk oligosaccharides
MyD88	Myeloid differentiation primary response 88
NF-κB	Nuclear factor kappa-B
OTU	Operational taxonomic unit
SL	Sialyllactose
SCFA	Short-chain fatty acids
TLR4	Toll-like receptor 4
TNF-α	Tumor necrosis factor-α
V/C	Villous height-to-crypt depth ratio
